# Novel combination therapy for platinum-eligible patients with locally advanced or metastatic urothelial carcinoma: a systematic review and network meta-analysis

**DOI:** 10.1007/s00262-024-03910-3

**Published:** 2025-02-01

**Authors:** Takafumi Yanagisawa, Keiichiro Mori, Akihiro Matsukawa, Tatsushi Kawada, Satoshi Katayama, Ekaterina Laukhtina, Pawel Rajwa, Fahad Quhal, Benjamin Pradere, Wataru Fukuokaya, Kosuke Iwatani, Renate Pichler, Jeremy Yuen-Chun Teoh, Marco Moschini, Wojciech Krajewski, Jun Miki, Shahrokh F. Shariat, Takahiro Kimura, European Association of Urology–Young Academic Urologists Urothelial Carcinoma Working Group (EAU-YAU), Takafumi Yanagisawa, Keiichiro Mori, Akihiro Matsukawa, Tatsushi Kawada, Satoshi Katayama, Ekaterina Laukhtina, Pawel Rajwa, Fahad Quhal, Benjamin Pradere, Wataru Fukuokaya, Kosuke Iwatani, Renate Pichler, Jeremy Yuen-Chun Teoh, Marco Moschini, Wojciech Krajewski, Jun Miki, Shahrokh F. Shariat, Takahiro Kimura

**Affiliations:** 1 Department of Urology, Comprehensive Cancer Center, Medical University of Vienna, Wahringer Gurtel 18-20, 1090 Vienna, Austria; 2 Department of Urology, The Jikei University School of Medicine, Tokyo, Japan; 3 Department of Urology, Okayama University Graduate School of Medicine, Dentistry and Pharmaceutical Sciences, Okayama, Japan; 4 Institute for Urology and Reproductive Health, Sechenov University, Moscow, Russia; 5 Department of Urology, Medical University of Silesia, Zabrze, Poland; 6 Department of Urology, King Fahad Specialist Hospital, Dammam, Saudi Arabia; 7 Department of Urology, La Croix Du Sud Hospital, Quint Fonsegrives, France; 8 Department of Urology, Comprehensive Cancer Center Innsbruck (CCCI), Medical University of Innsbruck, Innsbruck, Austria; 9 S.H. Ho Urology Centre, Department of Surgery, The Chinese University of Hong Kong, Hong Kong, China; 10 Department of Urology, San Raffaele Hospital and Scientific Institute, Milan, Italy; 11 Department of Minimally Invasive and Robotic Urology, Wrocław Medical University, Wrocław, Poland; 12 Division of Urology, Department of Special Surgery, The University of Jordan, Amman, Jordan; 13 Department of Urology, University of Texas Southwestern Medical Center, Dallas, TX USA; 14 Department of Urology, Second Faculty of Medicine, Charles University, Prague, Czech Republic; 15 Department of Urology, Weill Cornell Medical College, New York, NY USA; 16 Karl Landsteiner Institute of Urology and Andrology, Vienna, Austria

**Keywords:** Chemotherapy, Enfortumab vedotin, Immune checkpoint inhibitors, Metastasis, Urothelial carcinoma

## Abstract

Recent phase 3 randomized controlled trials (RCTs) demonstrate the promising impact of immune checkpoint inhibitor (ICI)-based combination therapies on locally advanced or metastatic urothelial carcinoma (UC). However, comparative data on the efficacy and toxicity of different ICI-based combinations are lacking. This study aims to compare the efficacy of first-line ICI-based combination therapies for locally advanced or metastatic UC using phase 3 RCT data. In November 2023, three databases were searched for RCTs evaluating oncological outcomes in patients with locally advanced or metastatic UC who were treated with first-line ICI-based combination therapies. Network meta-analysis (NMA) was conducted to compare outcomes, including overall survival (OS), progression-free survival (PFS), objective response rates (ORRs), complete response rates (CRRs), and treatment-related adverse events (TRAEs). Subgroup analyses were based on PD-L1 status and cisplatin eligibility. The NMA included five RCTs. Enfortumab vedotin (EV) + pembrolizumab ranked the highest for improving OS (100%), PFS (100%), ORR (96%), and CRR (96%), followed by nivolumab + chemotherapy. EV + pembrolizumab combination superiority held across PD-L1 status and cisplatin eligibility. In patients who are cisplatin-eligible, EV + pembrolizumab significantly improved OS (HR: 0.68, 95%CI 0.47–0.99) and PFS (HR: 0.67, 95%CI 0.49–0.92) compared to nivolumab + chemotherapy. Durvalumab + tremelimumab was the safest combination for severe TRAEs, and EV + pembrolizumab ranked second. Our analyses support EV + pembrolizumab combination as a first-line treatment for locally advanced or metastatic UC. Thus, EV + pembrolizumab may become a guideline-changing standard treatment.

**Supplementary Information:**

The online version contains supplementary material available at 10.1007/s00262-024-03910-3.

## Introduction

Locally advanced or metastatic stage 4 urothelial carcinoma (UC) is a lethal disease with a survival rate of less than 5% over a 5-year period [1, 2]. Despite the advent of novel agents, such as immune checkpoint inhibitors (ICIs), platinum-based chemotherapy has been the conventional treatment strategy for advanced or metastatic UC for over 30 years [1–4]. While ICIs, such as pembrolizumab and avelumab, have been the standard treatment after first-line platinum-based chemotherapy depending on the disease status [5, 6], the efficacy of ICI monotherapy or ICI-based combination therapy as a first-line treatment for advanced or metastatic UC remains debatable [7]. A recent meta-analysis, which synthesized the three phase randomized controlled trials (RCTs) (IMvigor130, KEY-NOTE 361, and DANUBE trials) assessing the efficacy of first-line ICI-based combination in platinum-eligible patients with advanced/metastatic UC, showed the oncologic benefit of ICI-based combination over chemotherapy alone [7], while each RCT failed to meet their primary endpoint, overall survival (OS) [8–10]. Most recently, the CheckMate 901 trial first demonstrated the OS benefit of adding nivolumab to gemcitabine-cisplatin in cisplatin-eligible patients with advanced/metastatic UC [11]. Moreover, the EV-302/KEYNOTE-A39 trial showed the outstanding survival benefit of the pembrolizumab plus enfortumab vedotin (EV) combination compared to chemotherapy in platinum-eligible patients with advanced/metastatic UC [12]. These findings will pave the way for new standard treatments for advanced UC. Data on the comparative efficacy among different combinations, coupled with the optimal identification of patients who will benefit from these combinations, will enrich clinical decision-making. There is currently no comprehensive information regarding the comparative efficacy and safety of different immunotherapy combinations as first-line treatments for advanced UC. Therefore, we conducted a systematic review and network meta-analysis (NMA) aimed at indirectly comparing the efficacy and safety, stratified by cisplatin eligibility and programmed death ligand 1 (PD-L1) expression status.

## Methods

The protocol of this study has been registered in the International Prospective Register of Systematic Reviews database (PROSPERO: CRD484628).

### Search strategy

**Fig. 1:**
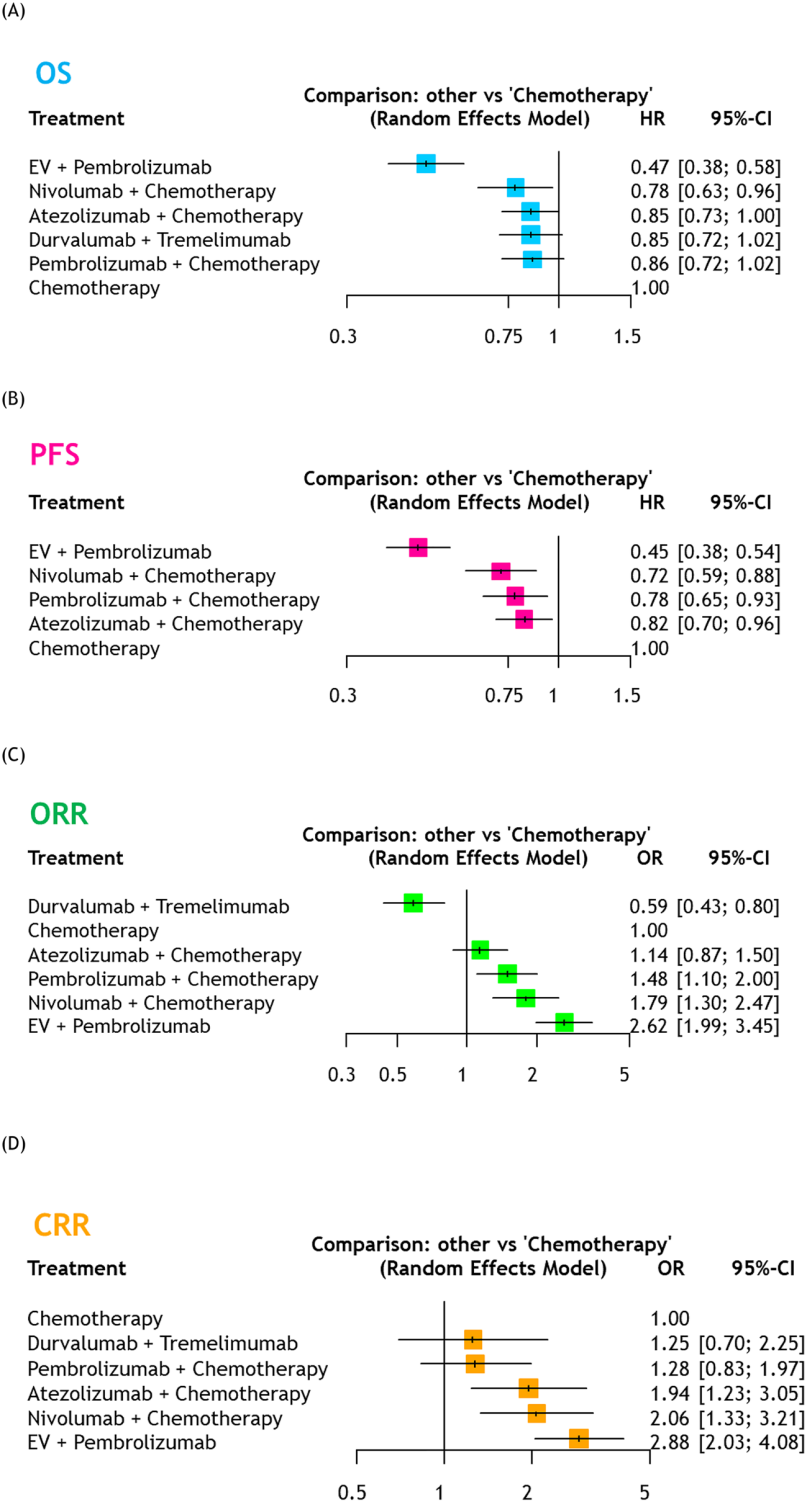
Forest plots showing the results of NMAs among overall population for OS(A), PFS(B), ORR(C), and CRR(D) in advanced/metastatic UC patients treated with first-line therapy *NMA* Network meta-analysis, *OS* Overall survival, *PFS* Progression-free survival, *ORR* Objective response rate, *CRR* Complete response rate, *UC* Urothelial carcinoma, *EV* Enfortumab vedotin, *HR* Hazard ratio, *CI* Confidence interval

This systematic review and NMA was performed following the guidelines of the Preferred Reporting Items for Systematic Reviews and Meta-Analyses (PRISMA) statement and PRISMA for NMA (Supplementary Table 1) [13, 14]. Three databases (PubMed^®^, Web of Science^™^, and Scopus^®^) were searched in November 2023 to identify studies assessing oncologic outcomes in advanced or metastatic UC patients treated with ICI-based combination therapies as a first-line treatment. The detailed search words were (urothelial carcinoma OR urothelial cancer) AND (metastatic OR advanced) AND (randomized), showing in Fig. 1 and Supplementary Appendix 1. Subsequently, abstracts presented at major conferences (i.e., the American Society of Clinical Oncology and the European Society for Medical Oncology) were searched to include trial updates. The main outcomes of interest were OS and progression-free survival (PFS). Additional outcomes of interest were objective response rates (ORRs), complete response rates (CRRs), and rates of treatment-related adverse events (TRAEs). Two investigators independently conducted the screening of the titles and abstracts. Potentially relevant studies were subjected to full-text review. Disagreements were resolved by establishing consensus among co-authors.

### Inclusion and exclusion criteria

Studies were included if they included patients with advanced/metastatic UC (Participants) and evaluated the efficacy of ICI-based combination therapies (Interventions) compared to the efficacy of chemotherapy (Comparisons) assessing their differential effects on OS, PFS, ORRs, CRRs, and/or rates of TRAEs (Outcomes) in RCTs (Study design). We excluded studies lacking original patient data, reviews, letters, editorial comments, replies from authors, case reports, and articles not written in English. Relevant references of eligible studies were scanned for additional studies of interest.

### Data extraction

Two authors independently extracted the relevant data on studies; the first author’s name, publication year, inclusion criteria, agents of the intervention and control arms, median age, the number of patients on each demographic (i.e., male, performance status, primary tumor, metastasis, high PD-L1 status, cisplatin eligibility, previous cancer therapy, and subsequent therapy), follow-up periods, ORRs, CRRs, TRAEs (any and severe defined as more than CTCAE grade 3). Hazard ratios (HRs) and 95% confidence intervals (CIs) for OS and PFS were extracted. In addition, we extracted detailed OS and PFS data stratified by cisplatin-eligibility and PD-L1 status as much as possible if the study showed the relevant data. All discrepancies were resolved by establishing consensus among co-authors. As the IMvigor130, DANUBE, KEYNOTE-361 trials failed to show any oncologic benefit of ICI monotherapy over chemotherapy alone, only data on ICI-based combinations versus chemotherapy were extracted [8–10, 15].

### Risk of bias assessment

The quality and risk of bias of eligible RCTs were evaluated based on the Cochrane Handbook for Systematic Reviews of Interventions risk-of-bias tool (RoB version 2) (Supplementary Fig. 1) [16]. Two authors independently conducted the risk-of-bias assessment of each study.

### Statistical analyses

NMAs using random-effect models for direct and indirect treatment comparisons across outcomes were conducted [17, 18]. Contrast-based analyses were applied with estimated differences in the log HR and the standard error calculated from the HRs and CI [19]. The relative effects were presented as HRs or odds ratios (ORs) and 95% CI [17]. The relative ranking of the different regimens was estimated in terms of OS, PFS, ORRs, CRRs, and TRAEs using the surface under the cumulative ranking (SUCRA) [17]. Subsequently, we carried out subgroup analyses for each outcome stratified by cisplatin eligibility and PD-L1 status. Network plots were made to depict the connectivity of the treatment networks. For meta-analysis, forest plots were utilized to analyze and summarize the HRs with a 95%CI. Heterogeneity among the outcomes of eligible studies in this meta-analysis was assessed using Cochrane’s Q test [20, 21]. A fixed-effects model was utilized to calculate the HRs for non-heterogeneous results [21]. All statistical analyses were performed using R version 4.3.0 (R Foundation for Statistical Computing, Vienna, Austria).

## Results

### Study selection and characteristics

Figure S1 (Supplementary Appendix 2) shows the PRISMA flowchart detailing the article selection process. We identified 1,564 publications in the initial literature search. After removing duplicates, 1,042 publications underwent title and abstract screening. We further excluded 1,019 publications based on our exclusion criteria, leaving 23 publications for full-text review. Ultimately, we identified five RCTs, encompassing 3,734 platinum-eligible advanced or metastatic UC patients treated with ICI-based combination therapy or chemotherapy [8–12, 15]. The details and patient demographics are presented in Table 1. All RCTs reported OS in the entire cohort and subgroups stratified according to PD-L1 status and cisplatin eligibility. The analyzed treatment regimens comprised atezolizumab + chemotherapy, durvalumab + tremelimumab, pembrolizumab + chemotherapy, nivolumab + chemotherapy, EV + pembrolizumab, and chemotherapy alone. The DANUBE trial did not include PFS data for durvalumab + tremelimumab, leading to its exclusion from PFS analyses [10]. The CheckMate 901 study exclusively focused on cisplatin-eligible patients [11]; therefore, we focused on subgroup analyses stratified by cisplatin eligibility.

**Table 1:** Study demographics, oncologic, safety outcomes of included RCTs assessing ICI-based 1st line treatment for advanced or metastatic UC

Study name and References		IMvigor130 Galsky et al. [8, 26]		DANUBE Powles et al. [6]		KEYNOTE-361 et al. [9]		CheckMate 901 van der Heijden et al. [11]		EV-302/KEYNOTE-A39 Powles et al. [12]	
Published year		2020/2023		2020		2021		2023		2023	
Treatment arm/Control arm		Atezolizumab + Chemotherapy	Chemotherapy	Durvalumab + Tremelimumab	Chemotherapy	Pembrolizumab + Chemotherapy	Chemotherapy	Nivolumab + Chemotherapy	Chemotherapy	EV + Pembrolizumab	Chemotherapy
Number of patients		451	400	342	344	351	352	304	304	442	444
Inclusion criteria		Platinum-eligible Previously untreated LA/mUC ECOG PS 0–2		Platinum-eligible Previously untreated LA/mUC ECOG PS 0–1		Platinum-eligible Previously untreated LA/mUC ECOG PS 0–2		Cisplatin-eligible Previously untreated LA/mUC ECOG PS 0–1		Platinum-eligible Previously untreated LA/mUC PD-(L)1 inhibitor naïve GFR ≥ 30mL/min ECOG PS 0–2	
Age, years, median (range)		69 (IQR: 62–75)	67 (IQR: 61–73)	68 (IQR: 60–73)	68 (IQR: 60–73)	69 (IQR:62–75)	69 (IQR: 61–75)	65 (range: 32–86)	65 (range: 35–85)	69 (range: 37–87)	69 (range: 22–91)
Male, n (%)		338 (75)	298 (75)	256 (75)	274 (80)	272 (78)	262 (74)	236 (78)	234 (77)	344 (78)	336 (76)
ECOG PS 2, n (%)		60 (13)	40 (10)	0	0	23 (7)	22 (6)	0	0	15 (3.4)	11 (2.5)
Primary tumor (bladder), n (%)		312 (69)	293 (73)	264 (77)	255 (74)	287 (82)	270 (77)	235 (77)	219 (72)	305 (69)	339 (76)
Metastasis, n (%)		401 (89)	366 (92)	329 (96)	323 (94)	327 (93)	328 (93)	261 (86)	269 (89)	ND	ND
Visceral metastasis, n (%)		259 (57)	239 (60)	268 (78)	266 (77)	259 (74)	252 (72)	**64 (21)	**64 (21)	318 (72)	318 (72)
Lymph node only, n (%)		80 (18)	67 (17)	73 (21)	77 (22)	81 (23)	94 (27)	ND	ND	103 (23)	104 (23)
High PD-L1 status*, n (%)		108 (24)	91 (23)	205 (60)	207 (60)	159 (45)	158 (45)	111 (37)	110 (36)	254 (58)	254 (58)
High PD-L1 definition		IC ≥ 5%		IC/TC ≥ 25%		TC CPS ≥ 10		TC ≥ 1%		TC CPS ≥ 10	
Cisplatin eligibility, n (%)		137 (30)***	136 (34)***	194 (57)	193 (56)	156 (44)	156 (44)	All		240 (54)	242 (55)
Previous systemic cancer therapy, n (%)		54 (12)	64 (16)	71 (21)	70 (20)	37 (11)	47 (13)	88 (29)	68 (22)	NA	NA
Subsequent therapy, n (%)		151 (33)	189 (47)	153 (45)	187 (54)	124 (35)	215 (61)	127 (42)	171 (56)	128 (29)	294 (66)
Subsequent ICI therapy, n (%)		38 (8)	98 (25)	18 (5.3)	111 (32)	23 (7)	169 (48)	25 (8)	123 (40)	7 (1.6)	260 (59)
ORR, n (%)	All patients	215/447 (48)	178/397 (45)	124/342 (36)	169/344 (49)	192/351 (55)	158/352 (45)	175/304 (58)	131/304 (43)	296/437 (68)	196/441 (44)
	High PD-L1 status	ND	37/84 (44)	96/205 (47)	56/113 (50)	91/159 (57)	73/158 (46)	ND		ND	
	Cisplatin-eligible	66/136 (49)	68/135 (50)	71/194 (37)	99/193 (51)	100/156 (64)	76/156 (49)	NA			
	Cisplatin-ineligible	149/311 (48)	110/262 (42)	53/148 (36)	70/151 (46)	92/195 (47)	82/196(42)				
CR, n (%)	All patients	63/447 (14)	31/397 (8)	27/342 (8)	22/344 (6)	53/351 (15)	43/352 (12)	66/304 (22)	36/304 (12)	127/437 (29)	55/441 (13)
	High PD-L1 status	ND	10/84 (12)	24/205 (12)	15/207 (7)	25/159 (16)	26/158 (17)	ND		ND	
	Cisplatin-eligible	25/136 (18)	14/135 (10)	ND		ND		NA			
	Cisplatin-ineligible	38/149 (12)	17/110 (6)								
Median OS, months		16.1 (14.2–18.8)	13.4 (12.0–15.3)	15.1 (13.1–18.0)	12.1 (10.9–14.0)	17.0 (14.5–19.5)	14.3 (12.3–16.7)	21.7 (18.6–26.4)	18.9 (14.7–22.4)	31.5 (25.4-NR)	16.1 (13.9–18.3)
HR for OS (95%CI)	All patients	0.85 (0.73–1.00)		0.85 (0.72–1.02)		0.86 (0.72–1.02)		0.78 (0.63–0.96)		0.47 (0.38–0.58)	
	High PD-L1 status	0.77 (0.55–1.09)		0.75 (0.60–0.94)		0.90 (0.69–1.18)		0.75 (0.53–1.06)		0.49 (0.37–0.66)	
	Low PD-L1 status	< 1%: 0.88 (0.68–1.15) 1–5%: 0.86 (0.68–1.08)		1.04 (0.80–1.36)		0.83 (0.66–1.05)		0.80 (0.62–1.04)		0.44 (0.31–0.61)	
	Cisplatin-eligible	0.76 (0.57–1.01)		0.86 (0.68–1.08)		0.88 (0.67–1.15)		0.78 (0.63–0.96)		0.53 (0.39–0.72)	
	Cisplatin-ineligible	0.89 (0.74–1.08)		0.86 (0.67–1.11)		0.84 (0.67–1.06)		NA		0.43 (0.31–0.59)	
Median PFS, months		8.2 (6.5–8.3)	6.3 (6.2–7.0)	3.7 (3.4–3.8)	6.7 (5.7–7.3)	8.3 (7.5–8.5)	7.1 (6.4–7.9)	7.9 (7.6–9.5)	7.6 (6.1–7.8)	12.5 (10.4–16.6)	6.3 (6.2–6.5)
HR for PFS (95%CI)	All patients	0.82 (0.70–0.96)		NA		0.78 (0.65–0.93)		0.72 (0.59–0.88)		0.45 (0.38–0.54)	
	High PD-L1 status	0.68 (0.49–0.95)				0.79 (0.60–1.04)		0.58 (0.41–0.81)		0.42 (0.33–0.53)	
	Low PD-L1 status	< 1%: 0.79 (0.61–1.03) 1–5%: 0.89 (0.70–1.13)				0.78 (0.62–0.98)		0.80 (0.62–1.02)		0.50 (0.38–0.65)	
	Cisplatin-eligible	0.73 (0.55–0.97)				0.67 (0.51–0.89)		0.72 (0.59–0.88)		0.48 (0.38–0.62)	
	Cisplatin-ineligible	0.84 (0.70–1.02)				0.86 (0.68–1.09)		NA		0.43 (0.33–0.55)	
Median follow-up, months		11.8/49****		41.2		31.7		33.6		17.2	
TRAEs, n (%)	Any	434/453 (96)	373/390 (96)	255/340 (75)	283/313 (90)	337/349 (97)	329/342 (96)	296/304 (97)	267/288 (93)	427/440 (97)	424/433 (96)
	Severe (> CTCAE grade3)	367/453 (81)	315/390 (81)	93/340 (27)	188/313 (60)	262/349 (75)	245/342 (72)	188/304 (62)	149/288 (52)	246/440 (56)	303/433 (70)
	Specific severe AEs (rates > 5%)	Anemia: 40%	Anemia: 38%	Increased lipase: 5%	Anemia: 20%	Anemia: 30%	Anemia: 33%	Anemia: 22%	Anemia: 18%	Skin reactions: 15.5%	NA
		Neutropenia: 37%	Neutropenia: 30%		Neutropenia: 21%			Neutropenia: 19%	Neutropenia: 15%	Peripheral neuropathy: 6.8%	
		Thrombocytopenia: 21%	Thrombocytopenia: 18%		Thrombocytopenia: 7%			Thrombocytopenia: 8%	Thrombocytopenia: 5%	Hyperglycemia: 6.1%	

*RCTs* Randomized controlled trials, *ICI* Immune checkpoint inhibitors, *UC* Urothelial carcinoma, *mUC* metastatic UC, *LA* Locally advanced, *ECOG* Eastern cooperative oncology group, *PS* Performance status, *IQR* Interquartile range, *IC* Immune cell, *TC* Tumor cell, *OS* Overall survival, *PFS* Progression-Free Survival, *HR* Hazard ratio, *CI* Confidence interval, *PD-L1* Programmed death ligand 1, *CPS* Combined positive score, IC0 [< 1%], *TRAE* Treatment-related adverse event, *NA* Not applicable, *ND* No data

*PD-L1 status were measured with different cell types and cut-off value depending on study

**Only the data on patients with liver metastasis

***According to ineligibility assessed by Galsky criteria, 42% and 44% of patients in both arms were cisplatin-eligible

****49 months since the last patient was randomlyassigned

### Risk of bias assessment

We presented the results of the risk of bias assessment for each domain in each eligible RCT (Supplementary Fig. 1). All eligible phase III RCTs had a low risk of bias. Using the AMSTAR2 checklist, the overall confidence in the results of this NMA was judged as “High” (Supplementary Appendix 1). [22]

### Network meta-analyses for oncologic and safety outcomes in overall platinum-eligible patients

Network plots for all oncological outcomes are shown in Supplementary Fig. 2. The results of the treatment rankings based on the SUCRA analysis are summarized in Table 2.

**Table 2:** Summary of results of treatment ranking analyses of network meta-analysis

	OS	PFS	ORR	CRR	TRAEs	
					Any grade	Severe
1. All patients	1.EV + Pem: 100% 2.Nivo + Chemo: 64% 3.Atezo + Chemo: 46% 4.Durva + Trem: 45% 5.Pem + Chemo: 42% 6.Chemo: 2.5%	1.EV + Pem: 100% 2.Nivo + Chemo: 64% 3.Pem + Chemo: 48% 4.Atezo + Chemo: 38% 5.Chemo: 0.2%	1.EV + Pem: 99% 2.Nivo + Chemo: 76% 3.Pem + Chemo: 62% 4.Atezo + Chemo: 39% 5.Chemo: 23% 6.Durva + Trem: 0%	1.EV + Pem: 96% 2.Nivo + Chemo: 71% 3.Atezo + Chemo: 65% 4.Pem + Chemo: 32% 5.Durva + Trem: 29% 6.Chemo: 7.0%	1.Durva + Trem: 99% 2.EV + Pem: 67% 3.Chemo: 48% 4.Atezo + Chemo: 43% 5.Pem + Chemo: 41% 6.Nivo + Chemo: 1.7%	1.Durva + Trem: 100% 2.EV + Pem: 80% 3.Chemo: 47% 4.Atezo + Chemo: 43% 5.Pem + Chemo: 26% 6.Nivo + Chemo: 4.2%
2. Patients stratified by PD-L1 status						
High PD-L1	1.EV + Pem: 99% 2.Durva + Trem: 58% 3.Nivo + Chemo: 58% 4.Atezo + Chemo: 53% 5.Pem + Chemo:27% 6.Chemo: 7.2%	1.EV + Pem: 98% 2.Nivo + Chemo: 68% 3.Pem + Chemo: 50% 4.Atezo + Chemo: 32% 5.Chemo: 1.5%	NA			
Low PD-L1	1.EV + Pem: 100% 2.Nivo + Chemo: 64% 3.Pem + Chemo: 56% 4.Atezo + Chemo: 50% 5.Chemo: 16% 6.Durva + Trem: 14%	1.EV + Pem: 100% 2.Pem + Chemo: 56% 3.Nivo + Chemo: 51% 4.Atezo + Chemo: 41% 5.Chemo: 2.3%				
3. Patients stratified by cisplatin eligibility						
Cisplatin-eligible	1.EV + Pem: 98% 2.Atezo + Chemo: 62% 3.Nivo + Chemo: 59% 4.Durva + Trem: 39% 5.Pem + Chemo: 36% 6.Chemo: 6.7%	1.EV + Pem: 98% 2.Pem + Chemo: 59% 3.Nivo + Chemo: 48% 4.Atezo + Chemo: 45% 5.Chemo: 0.4%	NA			
Cisplatin-ineligible	1.EV + Pem: 100% 2.Pem + Chemo: 54% 3.Durva + Trem: 47% 4.Atezo + Chemo: 41% 5.Chemo: 7.6%	1.EV + Pem: 100% 2.Atezo + Chemo: 51% 3.Pem + Chemo: 45% 4.Chemo: 4.6%				

*OS* Overall survival, *PFS* Progression-free survival, *ORR* Objective response rate, *CRR* Complete response rate, *TRAEs* Treatment related adverse events, *PD-L1* Programmed death ligand 1, *EV* Enfortumab vedotin, *Pem* Pembrolizumab, *Nivo* Nivolumab, *Atezo* Atezolizumab, *Durva* Durvalumab, *Trem* Tremelimumab, *Chemo* Chemotherapy, *NA* Not applicable

#### OS and PFS

In the analysis of OS, as shown in Fig. 1A, EV + pembrolizumab (HR: 0.47, 95%CI 0.38–0.58) and nivolumab + chemotherapy (HR: 0.78, 95%CI 0.63–0.96) resulted in improved OS compared to chemotherapy alone. The treatment rankings indicated that EV + pembrolizumab (100%) had the highest likelihood of improving OS, followed by nivolumab + chemotherapy (64%, Table 2 and Supplementary Fig. 3).

All ICI-based combinations improved PFS compared to chemotherapy alone (Fig. 1B), with EV + pembrolizumab showing the maximum PFS benefit (100%, Table 2 and Supplementary Fig. 3).

#### ORR and CRR

In comparison to chemotherapy, EV + pembrolizumab (OR: 2.62, 95%CI 1.99–3.45), nivolumab + chemotherapy (OR: 1.79, 95%CI 1.30–2.47), and pembrolizumab + chemotherapy (OR: 1.48, 95%CI 1.10–2.00) resulted in improved ORRs (Fig. 1C). Treatment rankings revealed that EV + pembrolizumab (99%) had the highest likelihood of improved ORRs, followed by nivolumab + chemotherapy (76%) and pembrolizumab + chemotherapy (62%, Table 2 and Supplementary Fig. 3).

For CRRs, EV + pembrolizumab (OR: 2.88, 95%CI 2.03–4.08), nivolumab + chemotherapy (OR: 2.06, 95%CI 1.33–3.21), and atezolizumab + chemotherapy (OR: 1.94, 95%CI 1.23–3.05) demonstrated improved CRRs (Fig. 1D). Treatment rankings indicated that EV + pembrolizumab (96%) had the highest likelihood of improving CRRs, followed by nivolumab + chemotherapy (71%) and atezolizumab + chemotherapy (65%, Table 2 and Supplementary Fig. 3).

#### TRAEs

Figure S2 (Supplementary Appendix 2) illustrates the forest plots of the ORs for any and severe TRAEs. In comparison to chemotherapy alone, durvalumab + tremelimumab was associated with lower likelihood of TRAEs, both any (OR: 0.32, 95%CI 0.20–0.50) and severe (OR: 0.25, 95%CI 0.18–0.35, Fig. S2A) TRAEs. Conversely, nivolumab + chemotherapy resulted in a higher likelihood of TRAEs, both any (OR: 2.91, 95%CI 1.27–6.68) and severe (OR: 1.51, 95%CI 1.09–2.10, Fig. S2B) TRAEs. EV + pembrolizumab did not result in a lower likelihood of any TRAEs (OR: 0.70, 95%CI 0.29–1.65) but did show a lower likelihood of severe TRAEs (OR: 0.54, 95%CI 0.41–0.72) compared to chemotherapy alone.

The treatment rankings indicated that durvalumab + tremelimumab had the highest safety profile concerning both any (99%) and severe (100%) TRAEs, followed by EV + pembrolizumab (67% and 80%, respectively, Supplementary Fig. 3).

### Network meta-analyses for oncologic outcomes stratified by PD-L1 status

Table 1 highlights the varied definitions and measurement methods of PD-L1 expression in each study, while all RCTs provided data on OS or PFS stratified by PD-L1 status or both.

In patients with high PD-L1 expression, EV + pembrolizumab (HR: 0.49, 95%CI 0.37–0.66) and durvalumab + tremelimumab (HR: 0.75, 95%CI 0.60–0.94) resulted in improved OS compared to chemotherapy alone (Supplementary Fig. 4). For PFS, EV + pembrolizumab (HR: 0.42, 95%CI 0.33–0.54), nivolumab + chemotherapy (HR: 0.58, 95%CI 0.41–0.81), and atezolizumab + chemotherapy (HR: 0.68, 95%CI 0.49–0.95) improved PFS compared to chemotherapy alone.

In patients with low PD-L1 expression, only EV + pembrolizumab improved OS compared to chemotherapy alone (HR: 0.44, 95%CI 0.31–0.61, Supplementary Fig. 4), while EV + pembrolizumab (HR: 0.55, 95%CI 0.38–0.65) and pembrolizumab + chemotherapy (HR: 0.78, 95%CI 0.62–0.98) improved PFS compared to chemotherapy alone.

### Network meta-analyses for oncologic outcomes stratified by cisplatin eligibility

**Fig. 2:**
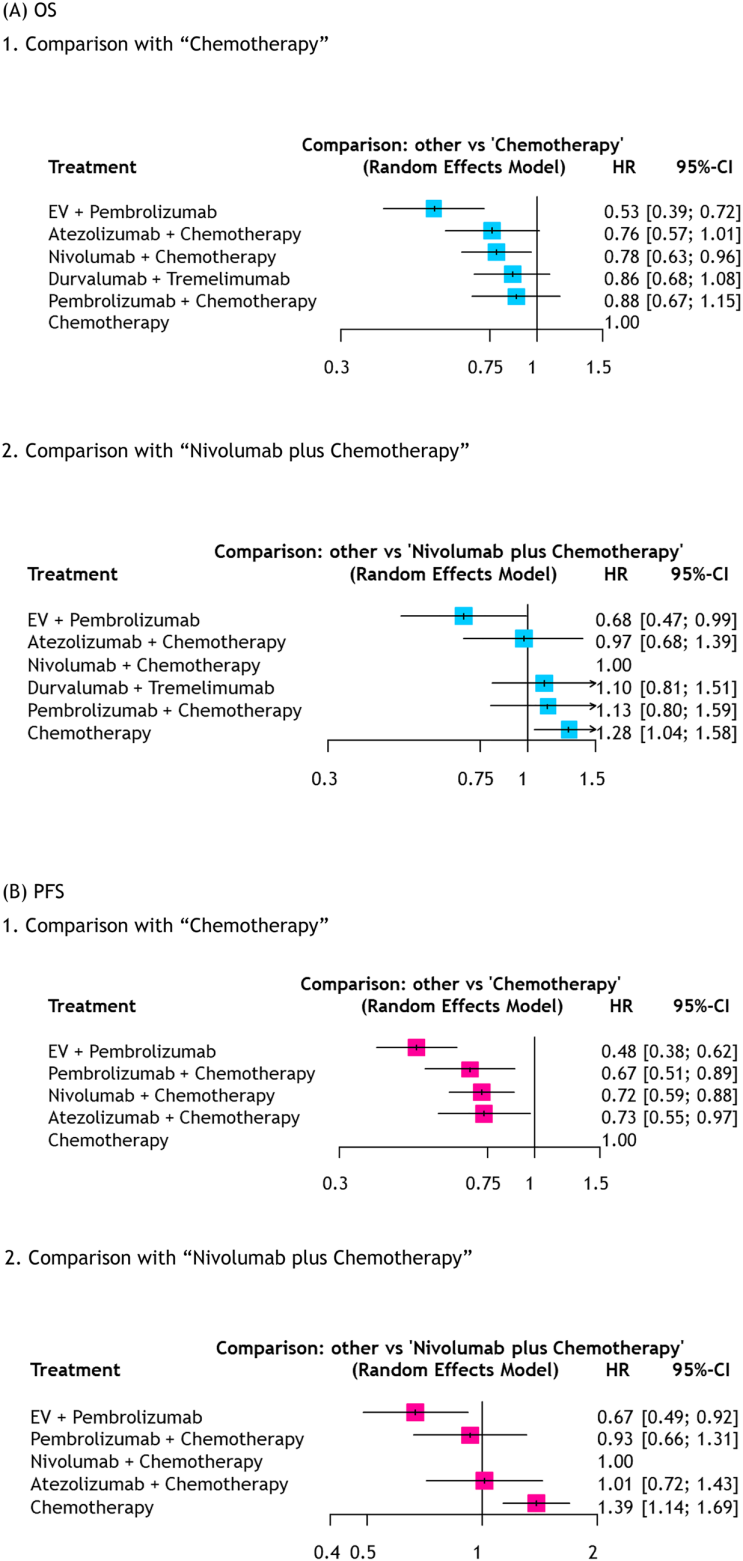
Forest plots showing the results of NMAs for OS(A) and PFS(B) in cisplatin-eligible patients with advanced/metastatic UC treated with first-line therapy *NMA* Network meta-analysis, *OS* Overall survival, *PFS* Progression-free survival, *UC* Urothelial carcinoma, *EV* Enfortumab vedotin, *HR* Hazard ratio, *CI* Confidence interval

All RCTs provided data on separate OS and PFS in patients with cisplatin-eligible. As shown in Fig. 2A, for patients with cisplatin-eligible, EV + pembrolizumab (HR: 0.53, 95%CI 0.39–0.72) and nivolumab + chemotherapy (HR: 0.78, 95%CI 0.63–0.96) resulted in improved OS compared to chemotherapy alone. Notably, when comparing to nivolumab + chemotherapy, EV + pembrolizumab significantly improved OS (HR: 0.68, 95%CI 0.47–0.99). For PFS, all four combinations resulted in improved PFS compared to chemotherapy alone (Fig. 2B). Similarly, compared to nivolumab + chemotherapy, EV + pembrolizumab significantly improved PFS (HR: 0.67, 95%CI 0.49–0.92). Treatment rankings are shown in Table 2 and Supplementary Fig. 6.

In patients with cisplatin-ineligible, only EV + pembrolizumab improved OS (HR: 0.43, 95%CI 0.31–0.59) and PFS (HR: 0.43, 95%CI 0.33–0.55, Supplementary Fig. 7).

### Meta-analysis for assessing the impact of adding ICIs to chemotherapy stratified by cisplatin-eligibility

Among five eligible RCTs, three RCTs assessed the impact of adding ICIs (i.e., atezolizumab, pembrolizumab, and nivolumab) to chemotherapy [8, 9, 11, 15]. Overall, adding ICIs to chemotherapy improved OS (HR: 0.83, 95%CI 0.75–0.92) and PFS (HR: 0.77, 95%CI 0.70–0.86) compared to chemotherapy alone (Supplementary Fig. 8). While the value of HRs were lower in patients with cisplatin-ineligible, there were no differences in terms of OS and PFS benefits between patients with cisplatin-eligible and those with cisplatin-ineligible (*p* = 0.4 and *p* = 0.085).

## Discussion

Our systematic review and NMA aimed to indirectly compare the efficacy and safety of treatments stratified by cisplatin eligibility and PD-L1 expression status, revealing several key findings. First, in the overall cohort, EV + pembrolizumab had the highest likelihood of improving OS, PFS, ORR, and CRR, followed by nivolumab + chemotherapy. Second, the superiority of the EV + pembrolizumab combination persisted across PD-L1 status and cisplatin eligibility. Notably, there was a significant improvement in OS and PFS with EV + pembrolizumab compared to nivolumab + chemotherapy in cisplatin-eligible patients. Third, while durvalumab + tremelimumab emerged as the safest combination for TRAEs, EV + pembrolizumab ranked second. In summary, our analysis not only demonstrated the overwhelming superiority of EV plus pembrolizumab in all evaluated oncological outcomes but also highlighted the regimen’s favorable safety profile.

EV is an antibody–drug conjugate (ADC) composed of the antibody enhortumab vedotin recognizing nectin-4 expressed on cancer cells and protease-cleavable linker-bound monomethyl auristatin E (MMAE), which disrupts microtubule formation [23]. By binding to nectin-4 expressed on cancer cells, the antibody forms an ADC-nectin-4 complex, allowing the complex to enter cancer cells and become available for lysosomal transfer. Thus, it is assumed that following cleavage of the linker by a protease, MMAE is released inside cancer cells and inhibits tubulin polymerization, leading to G2/M-phase cycle arrest and apoptosis of these cells. The mechanisms of synergy between the mitosis inhibitors MMAE and ICI have been accounted for by their ability to increase the release of dendritic cell-derived co-stimulants by MMAE, enhance T-cell capacity for stimulation, promote immunogenic cell death (ICD) (in vitro), exert immunomodulatory effects associated with the mouse xenografts involved, and promote the expression of immunogenic cell death-related genes in cancer cells [24, 25].

Moreover, the combination of ADC and ICI has been supported by several preclinical studies [23, 24]. An ADC is presumed to engage with the target antigen on cancer cells, undergo internalization, and subsequently release the cytotoxic payload, culminating in ICD. During this process, it is hypothesized that DAMPs are released in the tumor microenvironment (TME) and recognized by immature dendritic cells (DCs) via Toll-like receptors (TLR). This recognition, coupled with direct stimulation from the payload, enhances DC maturation, promotes migration to lymph nodes, and activates naïve T-cells [25]. Consequently, T-cells become available to infiltrate the tumor site, recognizing and attacking cancer cells. Meanwhile, the ICI unleashes an immune response against tumor cells, and the ADC activates the immune system through antibody-dependent cellular cytotoxicity (ADCC), antibody-dependent cell-mediated phagocytosis (ADCP) or complement-dependent cellular cytotoxicity (CDCC), or both [23]. In a study on nectin-4 overexpression in the human bladder cancer cell lines T24 and UM-UC-3, co-culturing these cells with nectin-4 non-expressing cell lines demonstrated the bystander effect of EV [29]. Moreover, cell lines exposed to EV showed evidence of an ER stress response induced via phosphorylation of Jun N-terminal kinase (JNK), which represents the initial signs of ICD [29]. Exposure to EV also led to the extracellular release of markers of ICD, such as the damage-associated molecular patterns ATP and HMGV1 [29]. Furthermore, an in vivo study using a T24 nectin-4 xenograft model demonstrated immune cell recruitment to cancer tissues and their activation [29]. Again, EV has been shown not only to induce ICD but also to enhance its effects when combined with an anti-PD-1 antibody in vivo [30]. Thus, while ADC has been demonstrated to induce tumor-specific acquired immunity, promoting T cell tumor infiltration, ICI has been shown to activate exhausted T cells, providing robust preclinical evidence for the use of combined ADC/ICI regimens.

Careful attention needs to be paid to differences in patient characteristics between the EV-302 and CheckMate 901 studies. First, the most significant difference lies in the patient populations involved; while the CheckMate 901 study included only cisplatin-eligible patients with gemcitabine plus cisplatin as the control treatment [11], the EV-302 study enrolled both cisplatin-eligible and cisplatin-ineligible patients with the control treatments with not only gemcitabine plus cisplatin but also gemcitabine plus carboplatin [12]. This suggests that the patient populations varied substantially in renal function between these studies. Additionally, differences in PD-L1 expression cut-off values and antibodies used further contribute to the variation. The EV-302 study likely enrolled more patients with high PD-L1 expression. Furthermore, in the CheckMate 901 study, only 3.3% of patients received EV after nivolumab plus chemotherapy, with approximately 20% of patients received ICI maintenance therapy after chemotherapy. In the EV-302 study, 33% patients are still on EV plus pembrolizumab, indicating a need for ongoing observation of improvements in OS over time in these patients. It should also be noted as a major difference between the two studies that in contrast to chemotherapy, nivolumab, and pembrolizumab whose duration were limited to 6 courses, 2 years and up to 35 courses, respectively, EV had no such restrictions in the EV302 study. However, with only a cisplatin-eligibility-stratified subgroup analysis reported in the EV-302 study, another subgroup analysis of EV plus pembrolizumab versus gemcitabine plus cisplatin is urgently needed. Despite these disparities, a comparison of OS in the control groups reveals similar outcomes gemcitabine plus cisplatin (18.4 months in the EV-302 study [cisplatin-eligible patients] versus 18.9 months in the CheckMate 901 study) with a comparable proportion of patients completing chemotherapy in the EV-302 study (55%) and the CheckMate 901 study (55%). This suggests a cogent rationale for comparing these studies. Additionally, platinum agents accounted for 86% of the treatment options following EV plus pembrolizumab, and ICI accounted for 88% of options following chemotherapy in the EV-302 study, closely reflecting real-world clinical practice.

Despite the comprehensive nature of this systematic review, some limitations must be considered. First, differences in patient characteristics at study enrollment among the RCTs, despite similar study designs, treatment lines, and target diseases, may have affected not only the oncological outcomes but also the AEs. Again, it should also be noted that the difference in the proportions of treatment options chosen after each regimen implemented as well as in their follow-up durations, which made it difficult to make adequate adjustments for potential confounders in the current NMA. The varying proportions of treatment options chosen after each regimen and differences in follow-up durations between the studies posed challenges in adequately adjusting for potential confounders in the current NMA. Second, the validity of the findings is contingent on the reporting quality and reliability of the reviewed trials, introducing potential bias and thereby limiting the validity of the findings. It is important to note that our present analysis was performed on a trial level. Third, while this study utilized indirect treatment comparisons of RCT outcomes, it does not intend to substitute head-to-head comparisons in clinical trials. Fourth, this comparison could not assess long-term durable benefits, e.g. cure rate in those exhibiting highly durable CR to nivolumab plus chemotherapy, suggesting that the regimen might prove curative in many CR patients. Besides, the duration of CR with EV-pembrolizumab was not available. Additionally, the current NMA may suffer due to its inability to control for sites of metastasis, a factor which may have accounted for differences in efficacy of the drugs evaluated, particularly given that nivolumab plus chemotherapy has now been shown to confer extremely high antitumor activity and benefit in those with lymph node-only metastasis in the CheckMate 901 trial [26], suggesting potential cures in this population and accumulation of further data from this subgroup is awaited. Fifth, the EV-302 trial results have yet to be published as a full paper, and we do not have access to the detailed data. Thus, the available data remain inconclusive as to how much drug dose led to the reported anti-tumor effects. Furthermore, the outcomes of the ongoing NILE trials may impact the role of ICI combination therapy in patients with metastatic UC. Sixth, the Javelin 100 trial contributed to maintenance therapy, establishing avelumab as the standard of care for patients without disease progression after first-line chemotherapy [6]. Therefore, the superiority of ICI combination therapy in the first-line setting needs yet to be evaluated in light of maintenance strategy. Further investigation is required to determine whether first-line combination therapy may take precedence over maintenance therapy, particularly given that those with PS 2 who account for a sizable proportion of patients encountered in clinical practice (i.e., those usually excluded from entry in RCTs) may be found unfit for EV + pembrolizumab and thus may be deemed candidates for maintenance therapy with avelumab. In addition, despite the impressive results with EV + pembrolizumab, other regimens may still play a role in the clinic due to comorbidities, such as neuropathy, poorly controlled diabetes mellitus, and liver dysfunction, particularly given the AE profile of EV + pembrolizumab (e.g., skin rash, neuropathy or lethal toxicities). Ongoing RCTs, including EV-304, KEYNOTE-B15, VOLGA, and EV-303/KEYNOTE 905 studies, are currently assessing the impact of EV plus pembrolizumab in perioperative settings, and the forthcoming results for this regimen in neoadjuvant and adjuvant settings are eagerly anticipated  [27].

## Conclusions

This study demonstrates that EV plus pembrolizumab exhibits potent and compelling anti-tumor effects as a first-line therapy for patients with locally advanced or metastatic UC. This robust evidence provides support for positioning EV plus pembrolizumab as a novel standard of care. There is a strong preclinical rationale for ADC/ICI combinations. Anticipating the ascendancy of EV plus pembrolizumab as the cornerstone of first-line treatment, several future considerations merit attention. These include determining viable treatment strategies for individuals deemed unsuitable for EV plus pembrolizumab, exploring available sequential therapies after EV plus pembrolizumab, and optimizing the overall utilization of EV.

## Supplementary Information

Below is the link to the electronic supplementary material.

Supplementary file1

Supplementary file2

## Data Availability

No datasets were generated or analysed during the current study.
