# Supplementary material for: Novel combination therapy for platinum-eligible patients with locally advanced or metastatic urothelial carcinoma: a systematic review and network meta-analysis
Source: Cancer Immunol Immunother. 2025 Feb 1;74(3):76. doi: 10.1007/s00262-024-03910-3 (PMC11787089; doi:10.1007/s00262-024-03910-3)

**Figure 1.** The Preferred Reporting Items for Systematic Reviews and Meta-analyses (PRISMA) flow chart, detailing the article selection process

**Identification of studies via databases and registers**

**Records identified through PUBMED, Web of Science, Scopus:**

**Search Query:**

(urothelial carcinoma) OR (urothelial cancer) AND (metastatic) OR (advanced) AND (randomized)

n=1,564

**Identification**

**Records excluded after title and abstract review** (n =1,019)

・Non-relevant according to inclusion criteria (n=859)

・Review article (n=101)

・Case reports (n=14)

・Letter/ Editorial comment (n=22)

・Other than English (n=23)

**Records screened after duplicates removed**

n =1,042

**Screening**

**Full-text articles assessed for eligibility**

n =23

**Records excluded after evaluation**

(n =17)

・Non-clear data regarding association between the systemic therapy and survival

**Eligibility**

**Included**

**Studies included in meta-analysis**

n=6 (5 RCTs)

**Figure 3.** Forest plots showing the results of NMAs among overall population for any (A) and severe (B) TRAEs in advanced/metastatic UC patients treated with first-line therapy

NMA: Network meta-analysis, TRAE: Treatment-related adverse event, UC: Urothelial carcinoma, EV: enfortumab vedotin, HR: Hazard ratio, CI: Confidence interval

(A)


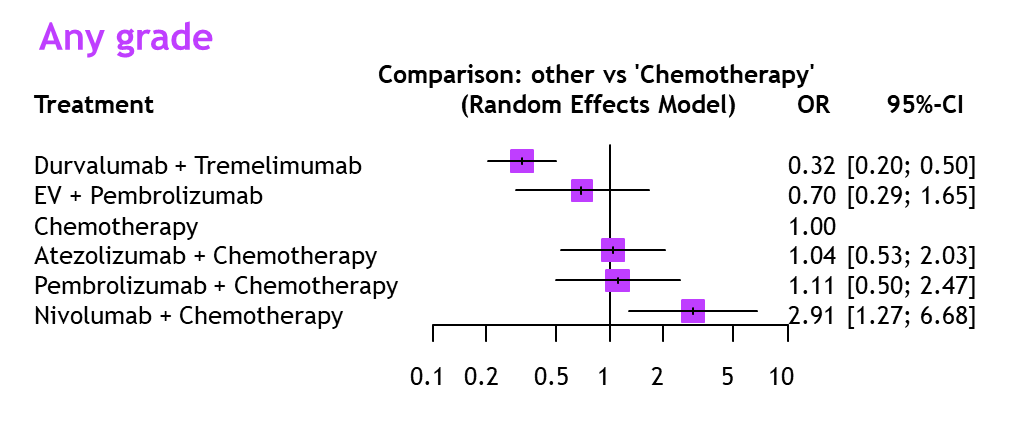


(B)


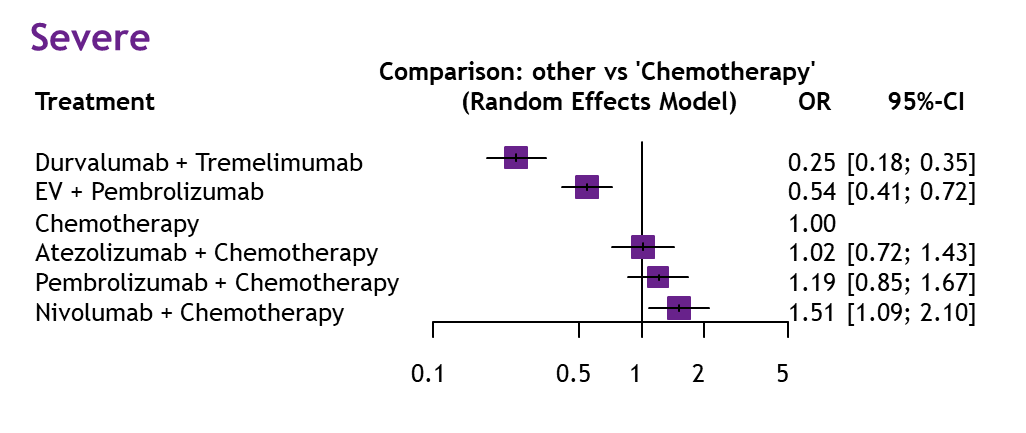

Supplement: Supplementary file 2 — Supplementary file2 [file 262_2024_3910_MOESM2_ESM.docx]
